# Guanxinning tablet for patients who switch from dual antiplatelet therapy to aspirin alone after percutaneous coronary intervention: study protocol for a cluster randomized controlled trial

**DOI:** 10.1186/s13063-017-2373-x

**Published:** 2018-02-07

**Authors:** Jingen Li, Jianqing Ju, Zhuo Chen, Jing Liu, Fang Lu, Rui Gao, Hao Xu

**Affiliations:** 1 Graduate School, Beijing University of Chinese Medicine, Beijing, 100029 China; 2 Cardiovascular Diseases Center, Xiyuan Hospital, China Academy of Chinese Medical Sciences, XiYuan CaoChang 1, Beijing, 100091 China; 3 Graduate School, China Academy of Chinese Medical Sciences, Beijing, 100700 China; 4 Institute of Clinical Pharmacology, Xiyuan Hospital, China Academy of Chinese Medical Sciences, XiYuan CaoChang 1, Beijing, 100091 China

**Keywords:** Coronary artery disease, Antiplatelet therapy, Guanxinning tablet, Chinese herbal medicine, Cluster randomized trial

## Abstract

**Background:**

One-year dual antiplatelet therapy (DAPT), generally aspirin in combination with a P2Y12 receptor inhibitor, has been a standard treatment for patients undergoing percutaneous coronary intervention (PCI). Prolonged DAPT has proven itself effective in further reducing cardiovascular events, yet with increased risk of bleeding. Thus, it is of great necessity to find an alternative drug that is as effective but safer and more economic than the P2Y12 inhibitors after termination of one-year DAPT.

**Methods:**

We will conduct a cluster randomized controlled trial in 3600 eligible post-PCI patients from 36 tertiary hospitals (100 patients per hospital) across mainland China. The hospitals served as clusters are randomized in a 2:1 ratio to Guanxinning tablet (GXNT) plus aspirin or aspirin alone for 12 months, with other conventional treatment applied in both groups. After the treatment period, all patients will be followed up for another 12 months. The primary outcome measure is composite cardiovascular events including cardiovascular death, non-fatal myocardial infarction, stent thrombosis, revascularization, ischemic stroke, and re-admission due to unstable angina. Secondary outcome measures are all-cause mortality, each individual component of the primary outcome measure, and stopping or reducing the rate of nitroglycerin administration. Adverse events, including bleeding, will be closely monitored during the whole trial period. In addition, a cost-effectiveness study of GXNT for the study population will be conducted along with this trial.

**Discussion:**

This trial aims to determine whether the addition of GXNT will further improve prognosis without increasing bleeding risk for patients with coronary artery disease who have switched from DAPT to aspirin alone after PCI. Completion of this clinical trial might provide a novel, promising, and safer alternative to P2Y12 inhibitors for prolonged antiplatelet therapy in post-PCI patients.

**Trial registration:**

Chinese Clinical Trial Registry, ChiCTR-IIR-17010688. Registered on 20 February 2017.

**Electronic supplementary material:**

The online version of this article (10.1186/s13063-017-2373-x) contains supplementary material, which is available to authorized users.

## Background

Millions of patients worldwide receive percutaneous coronary intervention (PCI) for treatment of coronary artery disease [1, 2], and treatment with dual antiplatelet therapy (DAPT) for 12 months after PCI has been considered standard care for preventing stent thrombosis and major adverse cardiovascular events (MACEs). Concerning the possibility of late or very late stent thrombosis, which is frequently associated with myocardial infarction, and may be fatal [3], researchers have conducted plenty of studies to explore the benefits and risks of prolonged (>12 month) DAPT [4–9]. Among those, the DAPT trial is the largest and sufficiently powered trial, and demonstrated that, compared with 12 months’ DAPT, an additional 18 months treatment with DAPT  further reduced the risk of stent thrombosis (hazard ratio 0.29, 95% confidence interval 0.17 to 0.48, *p* < 0.001) and MACE rate (hazard ratio 0.71, 95% confidence interval 0.59 to 0.85, *p* < 0.001), establishing the antithrombotic benefits of extended DAPT (>12 months) in post-PCI patients [9]. Later, a systematic review comparing extended DAPT (>12 months) with standard 12 month therapy also confirmed the benefits of extended DAPT, demonstrating that extended DAPT significantly reduced the risk of myocardial infarction (0.53, 95% confidence interval: 0.42 to 0.66) and stent thrombosis (0.33, 95% confidence interval: 0.21 to 0.51) [10]. However, all the antithrombotic benefits of extended DAPT are accompanied by increased bleeding risk, which has raised broad concerns. For example, in the DAPT trial [9], researchers found that 30 months of DAPT led to a 0.9% absolute increase in moderate to severe bleeding risk and a risk–benefit analysis showed that prolonged DAPT, annually, resulted in three fewer stent thromboses and six fewer myocardial infarctions at the cost of five more major bleeds per 1,000 patients. Notably, a recently published meta-analysis of 12 trials, comprising over 34,000 patients receiving different durations of DAPT, revealed that bleeding was an independent predictor of 1-year mortality and that longer duration of DAPT was associated with more bleeding-related deaths [11]. Besides, coronary artery disease itself is an exceedingly costly disease, costing a total of $316.6 billion annually in the USA [1], and prolonged application of the P2Y12 inhibitors will definitely add to the economic burden of both the nation and individuals. Thus, it is urgent to find an alternative drug that is as effective, but safer and more economic than P2Y12 inhibitors.

Traditional Chinese medicine has been used to treat coronary artery disease for quite a long time and is receiving increasing popularity worldwide for its efficacy and low cost [12, 13]. Guanxinning tablet (GXNT) is a traditional Chinese patent medicine and has been approved for marketing by the China Food and Drug Administration. It is developed, for convenience and better compliance, from the widely used Guanxinning injection, whose efficacy in reducing angina attacks and alleviating ST segment depression in the electrocardiogram has been broadly recognized and proved. Results of a meta-analysis involving 6064 patients with angina comparing Guanxinning injection versus control treatments (mainly nitroglycerin and other forms of traditional Chinese medicine), Guanxinning injection plus conventional treatment versus conventional treatment alone, and Guanxinning injection in combination with other forms of traditional Chinese medicine plus conventional treatment versus conventional treatment showed that Guanxinning injection was more effective than all controls in improving angina symptom and alleviating ST segment depression [14]. Guanxinning tablet, usually prescribed as an additional medicine to guideline-recommended standard medical treatment in China, comprises extracts from two well-established Chinese herbal medicines: Danshen (*Salvia miltiorrhiza*) and Chuanxiong (ligustrazine, *Ligustium Wallichii Franch*). Danshen, with tanshinones and phenolic acids as its main active compounds [15], has long been widely used for the prevention and treatment of cardiovascular diseases. An emerging experimental study and clinical trials demonstrated that Danshen and its active compounds could prevent atherosclerosis by inhibiting platelet aggregation, decreasing low-density lipoprotein oxidation, preventing monocyte adhesion to endothelium, and impeding smooth muscle cell migration and proliferation, macrophage cholesterol accumulation, and proinflammatory cytokine production [16, 17]. Chuanxiong, also a commonly used Chinese herbal medicine, together with its active compounds tetramethylpyrazine and ferulic acid could inhibit platelet aggregation and thrombosis [18, 19]. Moreover, preclinical studies indicated that GXNT is effective in inhibiting platelet aggregation, decreasing blood viscosity, and scavenging free radicals, which are all key pro-thrombotic factors [20]. Notably, no adverse events have been reported during the daily use of GXNT. Thus, it appears that GXNT is safe and effective, and may be a promising alternative to P2Y12 inhibitor for prolonged antiplatelet therapy in patients undergoing PCI. However, currently there is no adequately powered study assessing the safety and long-term efficacy of GXNT on clinically relevant outcomes for post-PCI patients.

Therefore, we designed this multicenter cluster randomized controlled trial to assess the long-term benefits, safety, and cost-effectiveness of GXNT for patients who switch from DAPT to aspirin alone after PCI.

## Methods

### Objectives and design

This study is a prospective, multicenter cluster randomized controlled trial aiming to explore whether the addition of GXNT to conventional treatment, including aspirin, will decrease MACEs without increasing bleeding risk for post-PCI patients who switch to aspirin monotherapy after the standard 12 months DAPT. An economic evaluation with 360 participants will be carried out alongside this trial in six of the 36 centers to assess the long-term cost-effectiveness of GXNT. The selection of the six centers takes both area and hospital type into account; three centers in Beijing and three centers in Hangzhou will be chosen. This study follows the international recommendations for interventional trials [21]. (See the SPIRIT checklist in Additional file 1.)

### Inclusion criteria

To be eligible to participate in the trial, patients should:

1. Be currently stable (without angina or with stable angina, defined as persistent and reproducible chest discomfort caused by myocardial ischemia and precipitated by a physical exertion that dissipates upon cessation of such an activity, within the last 3 months before screening) and have received standard DAPT (aspirin plus clopidogrel or prasugrel or ticagrelor) for at least 12 months after PCI
2. Have switched from DAPT to aspirin monotherapy for at least one month
3. Be diagnosed with the blood stasis pattern of traditional Chinese medicine, which is the most common traditional Chinese medicine syndrome of coronary artery disease; patients with any one of the following signs will be diagnosed as having the blood stasis pattern:
  - dark purple or dark red tongue, or petechia or ecchymosis
  - bluish-purple or dark color of the lips
  - varicosity or abnormal telangiectasia of the sublingual veins [22]
4. Be between 18 and 80 years of age
5. Provide written informed consent.

### Exclusion criteria

Patients will be excluded if they:

1. Have complications with serious heart failure (New York Heart Association class IV)
2. Have severe and uncontrolled high blood pressure (systolic blood pressure ≥ 180 mmHg or diastolic blood pressure ≥ 110 mmHg)
3. Have severe cardiopulmonary dysfunction, or serious primary disease of the liver, kidney, and hemopoietic system, or psychosis, or a malignant tumor
4. Have a high risk of bleeding, such as a hemorrhage history of vital organs (brain or upper gastrointestinal tract) within the past 6 months, low platelet counts, coagulation disorder, or current treatment with warfarin or other anticoagulant therapy
5. Are allergic to the study drugs (GXNT or aspirin) or its components
6. Are pregnant, planning to be pregnant or breast-feeding
7. Have previously been involved in another clinical trial in the past one month

### Settings and recruitment

Qualified drug clinical trial institutions or tertiary hospitals across mainland China have been invited, and the first 36 hospitals to agree to participate will be included. Each hospital is expected to recruit 100 eligible patients. Patients can be recruited by screening inpatients and outpatients of each hospital, through media advertising and social media or by screening a PCI database. Potential eligible patients will be screened and assessed by trained investigators, and eligible participants will be asked to provide written informed consent.

### Allocation

**Fig. 1:**
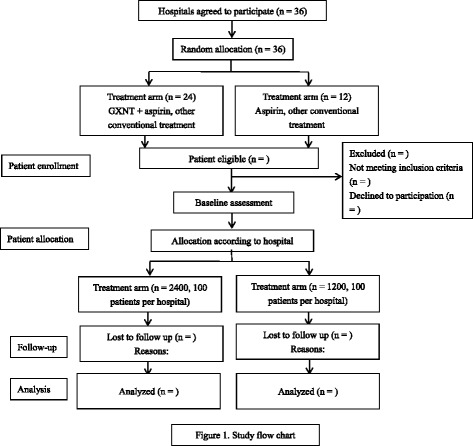
Study flow chart. GXNT, Guanxinning tablet

Thirty-six participating hospitals (clusters) with 3600 patients are firstly stratified by type of medication delivery (Western medical or traditional Chinese medicine hospitals), and then hospitals in each stratification are randomly allocated in a 2:1 ratio by a computer-generated, centrally controlled randomization schedule to treatment (24 hospitals with 2400 patients) or control (12 hospitals with 1200 patients) arms (see Fig. 1). All patients from one cluster will receive same intervention. Though the patients and caregivers are not blinded, the outcome assessors and data analysts are to be blinded to treatment allocation to minimize potential bias.

### Interventions

All recruited patients will continue to receive aspirin (100 mg once daily) and other conventional treatment including lipid-reducing, antihypertensive, or antidiabetic therapy [23]. Patients recruited from hospitals in the treatment arm will additionally receive GXNT (0.38 g/tablet, China State Food and Drug Administration approval number Z20150028) four tablets each time, three times daily for the first 6 months, and reduce to four tablets each time, twice daily for another 6 months, whereas patients from the controlled hospitals will only receive conventional treatment. At 12 months, patients in the treatment arm will stop GXNT treatment, and then all participants in both the treatment and control arm will continue aspirin monotherapy and other conventional treatment for another 12 months, until month 24. Any kind of other traditional Chinese medicine, including Guanxinning injection, is not allowed for all participants during the whole trial period.

### Outcome measures

The primary efficacy outcome measure is the composite cumulative incidence of MACE, including cardiovascular death, non-fatal myocardial infarction, stent thrombosis, revascularization, ischemic stroke, and re-admission for unstable angina during the treatment and follow-up periods. The secondary efficacy outcome measures are stopping or reducing the rate of nitroglycerin tablets and the respective cumulative incidence of all-cause death, cardiovascular death, non-fatal myocardial infarction, stent thrombosis, revascularization, ischemic stroke, and re-admission for unstable angina. Other efficacy outcomes include hypersensitivity C-reactive protein and platelet aggregation rate. Patients’ costs, the Seattle Angina Questionnaire rating, and health-related quality of life determined by the generic Short Form-36 (SF-36) questionnaire will be additionally assessed for cost-effectiveness analysis.

### Safety outcomes and compliance monitoring

The incidence of bleeding defined according to the Bleeding Academic Research Consortium definitions [24], any adverse events or serious adverse events, and compliance of the participants will be closely monitored and recorded during the whole trial period. If necessary, treatment will be ceased and patients will be withdrawn according to the nature of adverse events or serious adverse events. Compliance of the participants with the intervention is assessed by counting tablets of GXNT and reviewing tablet log records.

### Follow-up

**Fig. 2:**
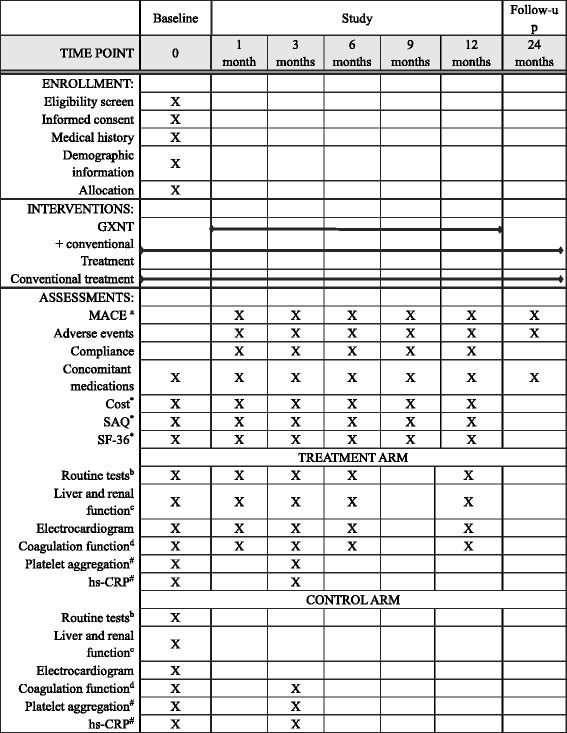
Trial schedule. ^*^The Seattle Angina Questionnaire (SAQ) and Short Form-36 (SF-36) will be only measured in the six hospitals selected for economic evaluation. ^#^The platelet aggregation and hypersensitivity C-reactive protein (hs-CRP) are to be tested in both control and treatment arm in one or two centers in Shanghai. ^a^Major adverse cardiovascular and cerebrovascular events (MACEs) include all-cause death, cardiovascular death, non-fatal myocardial infarction, stent thrombosis, revascularization, ischemic stroke, and rehospitalization for unstable angina. ^b^Routine tests include complete blood count, urinalysis, and stool tests. ^c^Includes levels of aspartate aminotransferase, alanine aminotransferase, alkaline phosphatase, gamma-glutamyl transpeptidase, total bilirubin, and direct bilirubin. ^d^Coagulation function includes levels of prothrombin time, thrombin time, international normalized ratio, activated partial thromboplastin time, and fibrinogen

After baseline visit, all participants will receive follow-up visits at 1, 3, 6, 9, 12, and 24 months, as scheduled (see Fig. 2 for SPIRIT figure). The primary and secondary outcome measures will be monitored during the whole study period. Most laboratory tests in the treatment arm will be tested at baseline and at 1, 3, 6, and 12 months, while in the control arm most tests will only be conducted at baseline. Platelet aggregation rate and hypersensitivity C-reactive protein level will be measured in both the treatment and control arm at baseline and in the third month in one or two selected centers in Shanghai to explore the potential effects of GXNT on platelet aggregation and inflammation.

### Sample size and data analysis

As little data on MACE rate in post-PCI patients who switch from DAPT to aspirin for about 12 months is available, we have to calculate the sample size according to the results of other studies. One registry trial reported that the annual rate of death or myocardial infarction was approximately 4.7%/year for patients who underwent successful implantation of at least one drug-eluting stent and did not received prolonged DAPT [25]. Another retrospective study indicated that the 2 year composite rate of mortality, reinfarction, and target vessel revascularization for patients undergoing primary PCI after acute myocardial infarction was 23% to 30% [26]. Though participants in our trial may have a lower rate of mortality, reinfarction, and target vessel revascularization than the retrospective study, taking other components into account, including stent thrombosis, ischemic stroke, and re-admission for unstable angina, we estimate the 12 month rate for the primary outcome measure in patients taking aspirin alone might be 21%, and that GXNT will reduce the incidence by 21%. A statistic power of 0.8 and a type I error rate of 0.05 (alpha) are chosen. According to a previous study, we assume the coefficient of intracluster correlation to be 0.002 [27] and we expect to recruit 90 patients from each cluster (hospital). Thus, a total of 3240 patients with 36 clusters (hospitals) will be needed. Considering a dropout rate of 10%, a minimum of 3564 patients is needed; for convenience of randomization, we decided to recruit 3600 patients (100 patients per hospital) and 36 hospitals (2400 patients and 24 clusters in the treatment arm, 1200 patients and 12 clusters in the control arm). Considering the uncertainty of the MACE rates in the study population, an interim analysis will be conducted when one-third of expected MACEs occur and the sample size will be adjusted according to the results of the interim analysis.

Data analysis will be conducted by Nanjing Medical University. All analyses will be performed on the individual level with adjustment for clustering and all available data from the dropouts will be included in the analysis up to the time of dropout where possible. All statistical hypothesis tests will be two-tailed and performed at a 5% significance level. Continuous variables will be presented as means and standard deviation, median or interquartile range. Categorical variables will be expressed as frequencies and percentages. Normality of relevant data will be examined using the Shapiro–Wilk test. Cluster-adjusted baseline differences between arms will be analyzed using the independent Student’s *t* test or the Mann–Whitney test for continuous variables and the Pearson *χ*^2^ test for categorical variables. All outcome measures, including the primary outcome measure, will be analyzed using a generalized linear mixed-effect model with the fixed effect being the intervention and the random effect being hospitals, to account for the potential correlation of outcomes within each hospital. Moreover, we will calculate the absolute rate reduction with 95% confidence interval as a crude estimate of the rate difference between the treatment and control groups by using the Mantel–Haenszel method. The number needed to treat for the primary outcome measure as the inverted absolute rate reduction will also be calculated. Efficacy outcomes are to be analyzed according to the intention-to-treat principle.

Safety outcomes will be assessed among all randomized patients except those who do not take any study medication or who have no safety records. The safety outcomes will be summarized and analyzed using the approach outlined.

Missing data will be accounted for by the modern imputation methods and all analysis will be conducted using SAS software version 9.4 (SAS Institute, Cary, NC, USA).

### Data management and monitoring

Data from all participating centers will be imported into the clinical data management system (http://www.xyedc.com/OldXYEDC/). Methods will be adopted to ensure data accuracy and completeness. First, all source documents and laboratory reports will be reviewed by the study investigator and data entry staff to assure data accuracy. Second, site monitoring will be conducted by a clinical research associate to confirm protocol compliance, ethical standards, regulatory compliance, and data quality. Third, computer logic checks will be automatically run to identify such items as missing and questionable data. Forth, less common and more complicated errors will be checked manually. Identified errors will be corrected to ensure data quality.

In addition, a data safety monitoring committee independent of the sponsor will be recruited to guarantee the quality of the data and safety of the participants. If unexpected serious adverse events occur, and the data safety monitoring committee and investigators believe that GXNT will do great harm to participants, or results of the interim analysis to be conducted by the data safety monitoring committee show no trends of benefit, this study will be terminated early. Finally, an independent clinical endpoint committee will adjudicate all MACEs within the trial period and is blinded to treatment allocation.

## Discussion

Completion of this clinical trial may provide a novel, promising, and safer alternative to P2Y12 inhibitors for prolonged antiplatelet therapy, which may further reduce MACEs for patients who have already received 12 months DAPT after PCI without increasing bleeding risk. GXNT is a promising Chinese patent medicine for coronary artery disease. Experimental studies have demonstrated that GXNT could increase the serum NO level and coronary blood flow [28], reduce platelet aggregation and blood viscosity, and protect the endothelium [20]. A recent study demonstrated that GXNT might inhibit thrombosis through different mechanisms, such as inhibiting the formation of free radicals and intervening metabolism of arachidonic acid [29]. These findings imply that GXNT is an effective treatment to depress platelet activity and can be used for patients with coronary artery disease undergoing PCI. In fact, GXNT is now widely used in patients after coronary stent implantation in China. However, there is insufficient evidence to show whether the long-term use of GXNT in patients who have received DAPT for at least 12 months after PCI can generate further cardiovascular benefits and whether it is cost-effective. All the findings and facts stated here serve as an impetus for large controlled trials to assess the effectiveness, safety, and cost-effectiveness of GXNT in patients with coronary artery disease after PCI. Thus, we designed this multicenter cluster randomized controlled trial, which will provide high-quality evidence for the use of GXNT in patients after coronary stent implantation who have switched from DAPT to aspirin monotherapy.

The study is designed according to international clinical trial principles. Data will be monitored periodically and quality control measures will be implemented strictly, which all ensure an objective and scientific assessment of GXNT. In addition, as recruitment is of great importance to generalizability, the adoption of a cluster randomized design and extensive recruitment of hospitals across mainland China greatly broaden representation and generalizability, albeit limited to China alone. Moreover, the cluster randomized design is less costly, time-saving and convenient to implement than conventional randomized controlled trials, and could also avoid potential contamination, which occurs when subjects in the control arm are exposed to the intervention [30].

However, some limitations existing in this study may be a cause for concern. Recruitment may be quite difficult for some hospitals, considering the targeting of eligible patients. However, we have made detailed recruitment strategies including screening the PCI database, using media advertising and social media, etc., and we believe that with substantial effort devoted to overcoming the problem, we could successfully complete the recruitment with the pre-specified sample size.

In conclusion, this study is designed to assess whether the addition of GXNT to aspirin and other conventional treatment in post-PCI patients after discontinuation of DAPT will further reduce MACEs without increasing the bleeding risk. Successful completion of this trial may provide a safe and effective alternative to P2Y12 inhibitors for long-term antiplatelet therapy.

## Additional file

Additional file 1: SPIRIT 2013 Checklist. (DOC 128 kb)

## Abbreviations

DAPT: Dual antiplatelet therapy
GXNT: Guanxinning tablet
hs-CRP: Hypersensitivity C-reactive protein
MACE: Major adverse cardiovascular and cerebrovascular event
PCI: Percutaneous coronary intervention
SAQ: Seattle Angina Questionnaire
SF-36: generic Short Form-36
